# Forecasting Diabetes Prevalence in California: A Microsimulation

**Published:** 2011-06-15

**Authors:** Lu Shi, Jeroen van Meijgaard, Jonathan Fielding

**Affiliations:** University of California Los Angeles (UCLA) School of Public Health; UCLA School of Public Health, Los Angeles, California; UCLA School of Public Health, Los Angeles, California

## Abstract

**Introduction:**

Setting a goal for controlling type 2 diabetes is important for planning health interventions. The purpose of this study was to explore what may be a feasible goal for type 2 diabetes prevention in California.

**Methods:**

We used the UCLA Health Forecasting Tool, a microsimulation model that simulates individual life courses in the population, to forecast the prevalence of type 2 diabetes in California's adult population in 2020. The first scenario assumes no further increases in average body mass index (BMI) for cohorts entering adolescence after 2003. The second scenario assumes a gradual BMI decrease for children entering adolescence after 2010. The third scenario builds on the second by extending the same BMI decrease to people aged 12 to 65 years. The fourth scenario builds on the third by eliminating racial/ethnic disparities in physical activity.

**Results:**

We found the predicted diabetes prevalence of the first, second, third, and fourth scenarios in 2020 to be 9.93%, 9.91%, 9.76%, and 9.77%, respectively. We found obesity prevalence for type 2 diabetes patients in 2020 to be 34.2%, 34.0%, 25.7%, and 25.6% for the 4 scenarios. Life expectancy in the third (80.56 y) and fourth (80.94 y) scenarios compared favorably with that of the first (80.32 y) and second (80.32 y) scenarios.

**Conclusion:**

For the next 10 years, behavioral risk factor modifications are more likely to affect obesity prevalence and life expectancy in the general population and obesity prevalence among diabetic patients than to alter type 2 diabetes prevalence in the general population. We suggest setting more specific goals for reducing the prevalence of diabetes, such as reducing obesity-related diabetes complications, which may be more feasible and easier to evaluate than the omnibus goal of lowering overall type 2 diabetes prevalence by 2020.

## Introduction

Prevalence of type 2 diabetes among California's adult population increased from 4.7% to 8.1% from 1994 to 2008 (1). Reducing the prevalence of type 2 diabetes has been a stated goal both for national initiatives like Healthy People 2010 (2) and state-specific initiatives like Healthy California 2010 (3). However, numerical goals for disease prevalence levels in these initiatives are often set according to the "better than the best" criterion (ie, the overall outcome for the entire future population should be at least better than the current level, as achieved by the best-performing subpopulation). In the case of diabetes prevalence, "better than the best" translates to achieving a prevalence of 2.5% by 2010 (3).

Aspiring to be "better than the best" is a good approach with regard to long-term goals for disease prevention. However, given the chronic nature of many medical conditions — particularly in the case of type 2 diabetes, for which there is no proven cure — 10 years may not be sufficient time for the overall prevalence to drop below the current level for the subpopulation with the lowest prevalence. Milstein et al (4) demonstrated by using systems dynamics simulations that lowering the nationwide prevalence of diabetes from 2004 to 2010 was overly ambitious, given the observed trend from 1980 to 2003. Even for behaviors like cigarette smoking, a scenario in which people frequently quit, a goal of "better than the best" has been shown to be unattainable within 10 years (5,6).

California's diabetes risk profile is different from that of the rest of the United States, in part because of the state's higher proportion of Latinos, higher proportion of people without a high school diploma, and younger average age (7-9). Therefore, establishing and evaluating a state-specific goal of lowering prevalence of type 2 diabetes is important. We used the UCLA Health Forecasting Tool (UCLA-HFT) to project the prevalence of type 2 diabetes in California for the year 2020 and examine the potential effect of risk factor modifications on the forecasted prevalence of diabetes. The objective of this study was to examine whether reducing the prevalence of type 2 diabetes by 2020 can be achieved by modifying obesity and physical activity rates in the California population.

## Methods

The UCLA-HFT is a microsimulation model calibrated to represent demographics and population health, including health behaviors and disease outcomes, for county-, city-, and state-level populations in the United States. Age, race, and sex are set as demographic predictors of one's diabetes incidence, whereas cigarette smoking, physical activity, and body mass index (BMI) are set as behavioral risk factors that affect both the diabetes onset for people without diabetes and the diabetes case fatality for people with diabetes. Details of this model have been described elsewhere (10).

We used the UCLA-HFT to forecast prevalence of type 2 diabetes among California's adult population under different scenarios in 2020 (Table 1). The first scenario (the baseline scenario) built on recent observations that rates of childhood overweight and obesity have been leveling off in California and nationwide (11,12), assuming no further increase in obesity rates for the cohorts entering adolescence (defined as being aged 12 to 17 years) after 2003, when obesity rates first started leveling. In other words, in this baseline scenario, all cohorts that reach age 12 years after 2003 have the same BMI distribution as the cohort that turned age 12 in 2003.

The second scenario (called "childhood BMI decrease") was built on the observation that a considerable decline in rates of overweight and obesity in California has occurred among young children but not yet among adolescents (11). This scenario assumed a constant annual decrease in the BMI for children entering adolescence after 2010, until the 12-year-olds in 2028 have the same BMI distribution as 12-year-olds in 1985. In other words, we modeled a gradual return of obesity rates to the 1985 level by assuming a small annual reduction of BMI for every new cohort of 12-year-olds. Thus, the mean BMI decline for each subsequent cohort entering adolescence from 2010 to 2028 will be equivalent to the annual increase in mean BMI that was observed from 1985 to 2003. In our model, this cohort effect was modeled as a trend in the mean of the inverse of BMI, following other published research (13,14), increasing by 3.1 × 10^-4^ annually for boys aged 12 and increasing by 4.5 × 10^-4^ annually for girls aged 12. This increase in the mean of the inverse of BMI translates into an annual decrease in BMI of approximately 0.19 kg/m^2^ for boys with an initial BMI of 25.0 kg/m^2^ and a decrease of 0.18 kg/m^2^ for girls with an initial BMI of 25.0 kg/m^2^. The rate of decrease is higher for those with higher BMI initially because we modeled a mean shift in the inverse of BMI.

The third scenario (called "childhood and adult BMI decrease") made a more optimistic assumption for obesity control, assuming that annual BMI decline occurs not only among new cohorts of 12-year-olds every year (as in the second scenario), but also among adults aged 18 to 65 years who are overweight or obese. This scenario was simulated by assuming an annual BMI decrease for overweight and obese people from 2010 to 2028. The average annual BMI decrease was further assumed to be equivalent to the average annual BMI increase from 1985 to 2003. In our model, this was implemented as a stochastic increase in inverse BMI for people with a BMI more than 25.0 kg/m^2^ in the year. The inverse BMI increase is normally distributed with a mean of 1.4 × 10^-4^ and a standard error of 0.6 × 10^-4^ for men and a mean of 2.0 × 10^-4^ and a standard error of 0.9 × 10^-4^ for women. This translates into a mean annual BMI decrease of 0.13 kg/m^2^ for men with an initial BMI of 30.0 kg/m^2^ and 0.18 kg/m^2^ for women with an initial BMI of 30.0 kg/m^2^, with a larger decrement for those with a higher initial BMI.

The fourth scenario (called "BMI decrease with increase in PA levels") built on the third scenario by further assuming an increase of physical activity levels such that racial/ethnic disparities in physical activity levels are eliminated, an objective specified by public health professionals (15). As estimated from the Behavioral Risk Factor Surveillance System of the Centers for Disease Control and Prevention, non-Hispanic whites (50%) are more likely to meet the federal guideline (16) of at least 150 minutes per week of moderate-intensity physical activity or 75 minutes per week of vigorous-intensity physical activity than other racial/ethnic minority groups (African Americans, 37%; Latinos, 37%; Asians, 40%) (17).

Under this fourth scenario, starting from 2011, for each sex-age stratum all racial/ethnic subpopulations achieve the same physical activity level as the most active subpopulation in 2010.

We compared 4 simulated outcomes across the scenarios: type 2 diabetes prevalence among adults, obesity prevalence among adults, obesity prevalence among people with diabetes, and life expectancy at birth.

## Results

In 2008, type 2 diabetes prevalence among the adult population in California reached 8.1% (1). In none of the simulated scenarios did type 2 diabetes prevalence decrease below this level. The predicted type 2 diabetes prevalence in 2020 for the baseline scenario and for scenarios 2, 3, and 4 was 9.93%, 9.91%, 9.76%, and 9.77%, respectively (Table 2).

According to the baseline scenario, California's adult obesity prevalence will rise to 30.8% in 2020. This increase was attenuated in scenarios 2, 3, and 4, the largest reduction occurring in the third scenario in which an overall decrease in BMI and an increase in physical activity levels was applied (10.8 percentage points below the baseline prevalence). Among people with diabetes, obesity prevalence was 34.2% in the baseline scenario and decreased in all subsequent scenarios.

Life expectancy at birth was calculated by using age-specific death rates for the California population in 2020. In the baseline scenario, life expectancy was predicted to be 80.32 years. In the second scenario, in which a reduction in BMI for adolescents was applied, a reduction in life expectancy was not realized. Scenarios 3 and 4 yielded a gain in life expectancy of 0.24 year and 0.62 year.

## Discussion

To the best of our knowledge, this is the first study to use microsimulation to forecast trends in type 2 diabetes prevalence. Although we modeled ideal intervention scenarios, we found that these scenarios did not yield appreciably lower prevalence estimates for type 2 diabetes by 2020, compared with the baseline scenario. Furthermore, none of these scenarios projected a diabetes prevalence lower than that of the actual 2008 prevalence of 8.1%. These findings are similar to insights by Jones et al (18) that a considerable delay exists between primary prevention and downstream improvements in diabetes outcomes. Even effective prevention approaches in lifestyle modification to reduce obesity only slowed the increase of type 2 diabetes prevalence, at least for the first 10 years.

Unlike the systems dynamics model by Jones et al (18) in which obesity reduction nationwide from 2006 could lead to a "tipping point" of diabetes prevalence in 2018, none of our scenarios predicted such a point by 2020 for California. In our model, the changing demographics of the state, the aging of the California population, and the increasing proportion of high-risk demographic groups in the state all contributed to the increase of type 2 diabetes prevalence. Given the simulated individual life courses in our model, the increase in obesity prevalence starting in the 1980s affects future diabetes incidence, as the younger cohorts, for which overweight and obesity began to be epidemic, enter their 50s and 60s when type 2 diabetes prevalence rises rapidly. Therefore, the positive effect of risk factor modifications manifested in the lowered obesity prevalence among the adult population and reduced complications of obesity and diabetes in the third and fourth scenarios but did not decrease diabetes prevalence below the actual 2008 level. Our model confirms the findings of a study by Jones et al (18) that a reduction in obesity prevalence translates very slowly into a reduction in type 2 diabetes prevalence. Incorporation of demographic transition adds another component to this and other simulations when substantial variations in risk factors and baseline disease rates exist across age and race subgroups.

Another reason that obesity and physical activity interventions are not forecasted to lower type 2 diabetes prevalence from the 2008 level of 8.1% is that these interventions not only help prevent diabetes but also reduce the complication rates for diabetic patients. A leaner and more active population of patients with type 2 diabetes will live longer (19-23), so the reduction in new cases due to prevention are offset by longer life in those with the disease. Moreover, although the prevention effect of behavioral changes is confined to type 2 diabetes, both of the risk factor reductions increase the survival of patients with type 1 and type 2 diabetes. This counterintuitive result, whereby behavioral improvement could increase the prevalence of an epidemic, is best illustrated by the fact that adding a physical activity increase to the "childhood and adult BMI decrease" scenario slightly increases the obesity prevalence among the general adult population (Table 2).

This study has limitations. First, our model does not consider the behavioral mechanism whereby a prediabetes or a gestational diabetes diagnosis may trigger lifestyle change and pharmacologic treatment that could considerably reduce the onset probability of type 2 diabetes among people most at risk. A major lifestyle change among people most at risk for type 2 diabetes could have more immediate effect on incidence reduction than a lifestyle change randomly distributed among the general population. Second, we discuss only the outcome of obesity prevalence among patients with diabetes when we consider the effect of lifestyle changes on the health status of this group, although lifestyle changes such as physical activity increase may improve patients' quality of life in ways other than BMI reduction. A more comprehensive outcome metric is needed to better account for the overall effect of behavioral modifications on the health status of patients with type 2 diabetes.

A continuing observed rise in type 2 diabetes prevalence among the adult population in the coming decade does not necessarily reflect unsuccessful prevention interventions. It takes longer than a decade for risk factor modifications of historically reasonable amplitude to decrease type 2 diabetes prevalence. The effects of behavioral risk factor modifications are more rapidly observed in outcomes such as obesity prevalence and life expectancy, as well as in the complications of obesity and type 2 diabetes. In the case of diabetes control, more specific goals such as improving early detection of diabetes cases (as specified in Healthy California 2010), decreasing gestational diabetes (24), and reducing obesity-diabetes complications may be more feasible and easier to evaluate than the omnibus goal of lowering type 2 diabetes prevalence by 2020.

## Figures and Tables

**Table 1:** Assumptions of Future Obesity and Physical Activity Trends for the 4 Simulation Scenarios, California

**Scenario**	**Assumption for Body Mass Index (BMI) Distributions**	**Assumption for Disparities in Physical Activity (PA) Levels**
1. Baseline	All cohorts that become aged 12 years after 2003 have the same BMI distribution as the cohort turning 12 years in 2003	Disparities in PA levels will exist in the population as they did before
2. Childhood BMI decrease	Cohorts that become aged 12 years between 2003 and 2010 have the same BMI distribution as the cohort turning 12 years in 2003. After 2010, there is an annual decrease in BMI for children entering adolescence, until the 12-year-olds in 2028 have the same BMI distribution as those in 1985	Disparities in PA levels will exist in the population as they did before
3. Childhood and adult BMI decrease	The same as in "Childhood BMI decrease" scenario, plus a decline in BMI (equal to calibrated annual BMI increase from 1985 to 2003) among every person aged 12 to 65 years after 2010, except for those who are underweight	Disparities in PA levels will exist in the population as they did before
4. BMI decrease with increase in PA levels	The same as in "BMI decrease 12 to 65" scenario	In 2011, all subpopulations achieve the same PA levels as the most active subpopulation in 2010 (ie, disparities are eliminated)

**Table 2:** Simulated Health Outcomes for the Adult Population of California Under 4 Scenarios in the Year 2020^a^

Health Outcome	**Scenario**			
	**1. Baseline**	**2. Childhood BMI Decrease**	**3. Childhood and Adult BMI Decrease**	**4. BMI Decrease With Increase in PA Levels**
Diabetes prevalence, %	9.93	9.91	9.76	9.77
Difference from baseline	NA	−0.02	−0.17	−0.16
Obesity prevalence, %	30.8	27.2	20.0	22.2
Difference from baseline	NA	−3.6	−10.8	−8.6
Obesity prevalence among people with diabetes, %	34.2	34.0	25.7	25.6
Difference from baseline	NA	−0.2	−8.5	−8.6
Life expectancy at birth, y	80.32	80.32	80.56	80.94
Difference from baseline	NA	0	0.24	0.62

Abbreviation: BMI, body mass index; PA, physical activity; NA, not applicable.

a The definitions of each scenario are given in Table 1
